# An Overview of the Skin Microbiome, the Potential for Pathogen Shift, and Dysbiosis in Common Skin Pathologies

**DOI:** 10.3390/microorganisms13010054

**Published:** 2025-01-01

**Authors:** Anita Smith, Roberta Dumbrava, Noor-Ul-Huda Ghori, Rachael Foster, James Campbell, Andrew Duthie, Gerard Hoyne, Marius Rademaker, Asha C. Bowen

**Affiliations:** 1 Healthy Skin Team, Wesfarmers Centre of Vaccines and Infectious Diseases, The Kids Research Institute Australia, Perth, WA 6009, Australia; 2 Perth Children’s Hospital, Perth, WA 6009, Australia; 3 School of Medicine, University of Notre Dame, Fremantle, WA 6160, Australia; 4 Sir Charles Gairdner Hospital, Perth, WA 6009, Australia; 5 Department of Dermatology, Fiona Stanley Hospital, Perth, WA 6150, Australia; 6 Division of Infection and Immunity, School of Biomedical Sciences, University of Western Australia, Perth, WA 6009, Australia; 7 Central Perth Skin Clinic, Perth, WA 6000, Australia; 8 Institute of Respiratory Health, QEII Medical Centre, Perth, WA 6009, Australia; 9 School of Health Sciences, University of Notre Dame, Fremantle, WA 6160, Australia; 10 Clinical Trials New Zealand, Hamilton 3204, New Zealand; 11 Division of Paediatrics, School of Medicine, University of Western Australia, Perth, WA 6009, Australia

**Keywords:** skin microbiome, atopic dermatitis, rosacea, hidradenitis suppurativa

## Abstract

Recent interest in the diverse ecosystem of bacteria, fungi, parasites, and viruses that make up the skin microbiome has led to several studies investigating the microbiome in healthy skin and in a variety of dermatological conditions. An imbalance of the normal skin flora can cause some skin diseases, and current culture techniques are often unable to detect a microorganism to further our understanding of the clinical–microbiological correlates of disease and dysbiosis. Atopic dermatitis and rosacea are presentations that GPs often manage that may have an infective or microbiological component and can be challenging to treat. We aim to discuss the implications of the skin microbiome including the impact of dysbiosis on conditions such as these. We will also discuss some clinical pearls for initial and future directions of the management of conditions such as atopic dermatitis, rosacea, and hidradenitis suppurativa. Further research using culture-independent techniques is needed for conditions involving microbial dysbiosis to advance our knowledge of skin disease pathophysiology and guide future management.

## 1. Introduction

The skin microbiome is a diverse ecosystem composed of bacteria, fungi, and viruses; however, compared to other body sites, it has a very low biomass due to its nutrient-poor, exposed, and dry environment compared to the respiratory or gastrointestinal tracts [1,2]. The skin microenvironment has distinct physical–chemical properties of three major types, sebaceous (oily), dry, and axillae/follicular (moist), across the surface of the body and can therefore pose significant challenges to scientists in accurately sampling and characterising the microbial composition within these regions. Skin microbiome studies to inform the diagnosis and treatment of common dermatoses have focussed on bacterial species to date. Whilst viruses and fungi may contribute, our review will predominantly feature bacterial understandings that aid in diagnosis and treatment.

Over the past two decades, there have been numerous studies highlighting the importance of the skin microbiome in health and disease. The Human Microbiome Project Consortium found that the diversity and abundance of each habitat’s signature microbes among healthy subjects vary widely [3]. Further studies provided a framework for human microbiome research [4]. Oh et al. [5] identified strain-level variation in dominant species on the skin as heterogenous and multiphyletic, forming a foundation for human disease studies investigating inter-kingdom interactions, metabolic changes, and strain tracking.

Specifically in regard to eukaryotic organisms (such as *Malassezia* and *Demodex*) in the skin, these are reduced in abundancy compared to bacteria. *Demodex* predominantly resides in hair follicles and is an arachnid-genus, eight-legged mite [6]. Demodex are sebum-consuming mites (obligate human ecto-parasites), which are typically found in facial regions classically associated with rosacea. In cases of rosacea, skin samples have demonstrated higher frequencies of the demodex mite species when compared to control skin [7,8]. Research has also proposed that *Demodex* mites and their associated bacteria upregulate proteases that are linked to the further dysregulation of the cutaneous innate immune response [9]. In terms of the fungal components of the skin microbiome, these are unique in that there is predominantly *Malassezia*, a single fungus, in the skin mycobiome. It has been suggested that given this lack of diversity, *Malassezia* may outcompete other fungi from living on the skin [10]. Hypersensitivity to *Malassezia furfur* (yeast found naturally on the skin of humans) can result in a flare of atopic dermatitis on the head and neck, with *Malassezia*-directed treatment controlling the disease. Systematic reviews of the skin microbiome in patients with atopic dermatitis have found that there is a depletion of *Malassezia* spp. and high non-*Malassezia* fungal diversity [11]. Other systematic reviews [12] on seborrheic dermatitis have shown that the predominant fungi on the face and scalp were predominantly the fungi of *Ascomycota* and *Basidiomycota*. Additionally, there was an increased ratio of *Malassezia restrica*/*Malassezia globosa* in the setting of seborrheic dermatitis.

Currently, there is little information regarding the skin virome. Studies have described eukaryotic DNA viruses to be unique not to the site, but to the individual [13].

Most available protocols for microbial characterisation are based on those originally developed to analyse the high-biomass, high-diversity gut microbiome [14]. Low-biomass samples are susceptible to contamination from environmental sources in comparison to samples with a high deoxyribonucleic acid (DNA) microbial biomass, for example, faecal samples that are less likely to have issues with contamination during processing leading to false positives that have a reduced likelihood of other biases [15,16,17]. Until as recently as twenty years ago, methods of investigating human skin microbes relied primarily on culture-based techniques [1].

These initial culture studies found that the main skin bacterial genera included *Staphylococcus*, *Cutibacterium* (formerly *Propionibacterium*), *Corynebacterium*, and fungi such as *Malassezia* [1,18]. The challenge with traditional culture-based methods in a low-biomass environment is that not all microbes on the skin are able to be grown via culture techniques, creating a sampling bias, with the microbial richness of the skin being underestimated [19] and with some microbes not surviving once removed from the skin microenvironment [1]. There are several advantages of utilising 16S ribosomal ribonucleic acid (rRNA) gene sequencing techniques, including (i) the ability to reveal the presence of a large number of individual bacterial phyla; (ii) the ability to study the microbiome of particular skin diseases; (iii) the low cost compared to other sequencing methods; and (iv) the ability to avoid sequencing host DNA [19]. However, molecular techniques (e.g., amplicon-based) used for microbiome analysis are also limited in that short-read sequences are unable to provide accurate information about species or strains of microorganisms on the skin [1] and are highly dependent on the sampling and DNA extraction methods used. Other challenges include the multiple layers associated with the skin and the uneven species distribution on its surface [14].

Several sampling methods for investigating the skin microbiome have been described in recent years [20,21,22]. Cotton swabs and skin scrapings give rise to comparable skin microbiota profiles, representative of those obtained with skin biopsies, a technique often used in dermatology clinical practice to further evaluate for deeper skin infection and disease pathogenesis [20]. Tape stripping and scraping have also been reported in the literature but are suboptimal for skin microbiome analysis [22]. The use of adhesive patch sampling has been reported to be effective, well tolerated and non-invasive [23]; however, adhesive patch-based skin biopsy devices are difficult to procure and are not currently commercially available for clinical use. More invasive approaches including skin punch biopsies have been used to analyse the follicular skin microbiome using 16S rRNA and 18S rRNA sequencing [24]. The disadvantages of punch biopsies include that they are an invasive procedure that usually requires suturing, can leave a scar, which may be problematic if one wants to sample sites on the face (forehead, nose), and may not be appropriate for sensitive sites (such as axillae or the groin) [25]. Bjerre et al. previously compared flocked swabs vs. skin scrapings in adults, reporting that 99.3% of the sequences overlapped [14].

Further research using culture-independent techniques are needed for conditions involving microbial dysbiosis and to advance our knowledge of skin diseases, wound healing, and sepsis prevention. Dysbiosis describes the changes that occur in the microbiota, which promote the overgrowth of pathogenic species. The means by which local species establish specific niches on the skin and how they interact and alter the relative success of specific microbes represent a possible explanation for dysbiosis. Such changes are implicated in a range of skin conditions, several of which are commonly encountered in general practice, including atopic dermatitis, rosacea, and hidradenitis suppurativa, whereby antibiotics are commonly prescribed in management yet are often ineffective. Specifically, these three diseases have been chosen to represent different skin zones (e.g., sebaceous (rosacea), follicular (hidradenitis suppurativa), and dry (eczema)). Studies have shown that during a flare of atopic dermatitis, there is a decrease in bacterial diversity and an increase of approximately 35–90% in the proportion of the bacteriome made up of *Staphylococcus* spp. [26]. In hidradenitis suppurativa, studies have reported an increased (relative) abundance of certain anaerobic bacteria (such as *Peptoniphilus* spp., *Prevotella*, and *Porphyromonas*) in lesional skin in contrast to control or non-lesional skin. The relative abundance of anaerobic bacteria and the increase in the diversity of bacteria were also shown to correlate positively with the severity of hidradenitis suppurativa [27].

Compared to cotton swabs, flocked swabs have been shown to generate superior DNA extraction yields and are more suitable for direct polymerase chain reaction (PCR) [28,29,30]. Manus et al. [21] analysed 16S rRNA bacterial gene sequencing from swab samples taken from the axilla, hand, and forehead of 47 infants and found that the bacterial diversity and composition were shaped by skin site, age, socioeconomic factors, and household composition. The tip of a flocked swab is like a brush, allowing more surface area compared to cotton swabs and an ability to collect more material. The brush-like tip also enables the superior specimen collection and release of DNA during testing. To date, there is no established standard sampling method that produces unbiased results for skin microbiome studies.

Overall, further studies to optimise the molecular detection of bacteria from skin with standardised methods for sampling [1] are required to inform a broader understanding of skin health and skin disease and the complexity of its role in dysbiosis.

## 2. Methodology

A literature search using a narrative review style was performed to identify relevant articles to provide an overview of the skin microbiome, the natural resistance of skin, commensal organisms at different sites, and evidence to further outline the role of dysbiosis in atopic dermatitis, rosacea, and hidradenitis suppurativa.

### 2.1. Natural Resistance of Skin to Infection Including Barrier and Innate Defences

The skin functions as a physical barrier preventing infection, whilst also allowing a habitat for commensal organisms [31,32]. The skin constantly encounters pathogens, and to avoid infection, the dermis and epidermis have developed multiple innate defences such as antimicrobial peptides, including β-defensins, skin neuropeptide (substance P), and cathelicidins [31,32]. Many of these peptides have anti-bacterial, anti-viral, and anti-fungal activity in part due to their structural elements that allow the disruption of the microbial membrane whilst allowing the human cell membranes to remain intact [32]. Some peptides have a specific role in normal skin against certain microbes, whilst other peptides act when the skin’s barrier is damaged [33]. For example, cathelicidin peptides are increased and abnormally processed in rosacea [34] and in atopic dermatitis; a decreased expression of antimicrobial peptides can lead to an increased infection risk. Other aspects of the skin’s host defence include various cells such as natural killer cells, neutrophils, Langerhans cells, and lymphoid cells.

### 2.2. Commensal Organisms at Different Sites

**Figure 1:**
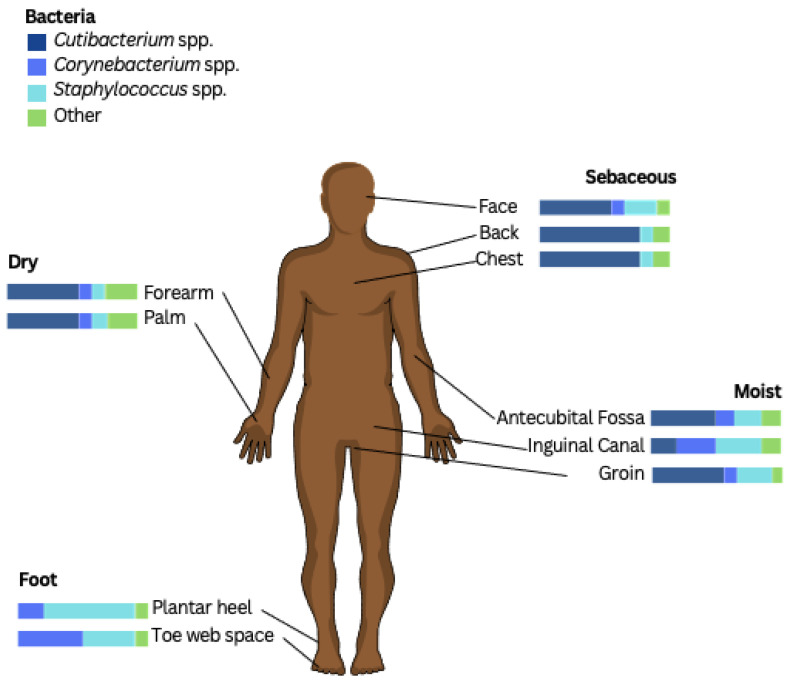
Skin microbial communities by the microenvironment of the skin [27,35]. Four sites are shown to represent the major microenvironments of the skin: face (forehead) (sebaceous/oily); antecubital fossa (moist); volar forearm (dry); and toe web space (foot). Bar graphs represent consensus relative abundances of the bacteria. The bacterial species *Cutibacterium* spp., *Staphylococcus* spp., and *Corynebacterium* spp. are displayed in bar charts to highlight relative abundance, with colours identified in the legend. Unlabelled species are grouped as ‘Other’. Figure adapted from [36]. Adaptation and reproduction of figure permitted by Creative Commons Attribution 4.0 International License, available from http://creativecommons.org/licenses/by/4.0/ (accessed on 26 December 2024).

The skin microbiome varies depending on moisture content, pH, temperature, and sebaceous gland concentration, in addition to other factors such as the exogenous environment and host genetics [32]. These can be represented by the sebaceous (oily) zone (e.g., forehead), the dry zone (e.g., volar forearm), and the moist zone (e.g., antecubital fossa, axilla). Figure 1 highlights the microbiome differences throughout these zones [35].

The microbiota are involved not only as commensal microorganisms but also in epithelial health and immune modulation [32]. Further research into these recently described roles has the potential to allow greater insight into the pathophysiology of skin conditions such as atopic dermatitis, as well as into the role of antimicrobial and promicrobial therapeutics such as probiotics [37].

Skin microbiome studies sample multiple sites to allow for differences in the zones of the skin. The most commonly employed sites used to sample and model the microbial community are (1) wet/non-oily (antecubital fossa); (2) dry/non-oily (volar forearm); (3) wet/oily (face-cheek/forehead); and (4) wet/oily (scalp) [38,39]. The toe web space is unique but minimally investigated (Reynolds, 2023) [40]. The results of a study conducted by our group [39] showed that in sampling the skin microbiome of three body sites, namely, the cubital fossa, cheek, and axilla, there was marked interpersonal variability, with each body site showing different taxa for each participant. In addition, this same study also showed that the skin microbiome was relatively stable over longitudinal sampling, maintaining temporal stability.

### 2.3. Pathogenicity of Bacteria and Potential for Commensal–Pathogen Shift

The main species of skin bacteria are *Cutibacteria*, *Corynebacteriae*, and *Staphylococci* [41]. There is a dynamic and rich interplay between these commensal organisms, many of which can modulate pathogenicity. Some *Cutibacterium* spp., for example, promote the virulence of *Staphylococcus aureus* [42]. Others, such as *Corynebacterium striatus*, have a “nurturing effect” of sorts, changing *S. aureus* from a pathogen into a commensal [43]. Other species such as *Corynebacterium accolens* act indirectly by making the local environment inhospitable for *Streptococcus pneumoniae* [44].

### 2.4. Dysbiosis in Atopic Dermatitis

Atopic dermatitis (AD) is a chronic inflammatory condition caused by an impaired skin barrier, dysregulated immunity, and microbial dysbiosis of the skin. The skin microbiome plays a critically important role in epidermal homeostasis, with dysbiosis in the microbiome being a contributing factor in the pathogenesis of atopic dermatitis [45]. The most prevalent organism isolated in areas of active eczema is *S. aureus*, which has also been shown to correlate with increased eczema flares [37]. A longitudinal study conducted in paediatric populations with eczema found that an increased total quantity of *S. aureus* correlated with greater disease severity during AD flares [37]. Hypersensitivity to *Malassezia furfur* (yeast found naturally on the skin of humans) can result in a flare of AD on the head and neck, with *Malassezia*-directed treatment controlling the disease. In addition, superantigens have also been implicated, with the proposed role of superantigens being that they promote the development of the Th2 immune response. In atopic dermatitis, up to 65% of *S. aureus* strains that colonise patients with atopic dermatitis have exotoxins with superantigenic properties [46].

Over 90% of patients with atopic dermatitis are colonised with *S. aureus* on their skin, in comparison to 5% of patients without atopic dermatitis, which has been proposed to reflect the decreased antimicrobial peptides (e.g., defensins, cathelicidins), disrupted acid mantle, and altered cytokine profile of skin in atopic dermatitis [47]. Studies have shown that during a flare of atopic dermatitis, there is a decrease in bacterial diversity and an increase of approximately 35–90% in the proportion of the bacteriome made up of *Staphylococcus* spp. [26]. Research has also shown that clinical improvement in atopic dermatitis correlates with the normalisation of the microbial population [48].

From a management point of view, there has been evidence to suggest that a multi-modal approach restores the skin bacteriome and reduces disease severity in AD. The use of topical corticosteroids, antimicrobials, and bleach baths in combination decreased the colonisation of *S. aureus* species and promoted further diversity in skin microbiota [49]. By using a variety of therapeutic interventions (Table 1), management may be able to alter the dysbiotic bacteriome in AD and restore it back to equilibrium.

### 2.5. General Skin Care Measures for Atopic Dermatitis

Bath or shower once a day using warm (not hot) water and keep it short (5–10 min).

Avoid using soap.

A bath oil can be added to the bath and a soap-free wash can be used if required.

Care must be taken with bath oil use in older children as it can make the bath very slippery.

The use of non-soap cleansers is recommended (i.e., soap-free wash or a soap substitute).

After bathing/showering, pat-dry the skin and apply moisturiser over the whole body and face.

Emollients (such as lanolin and glycerol stearate) are products used to smooth and soften the skin.

Avoid scratching the skin and keep the nails trimmed short.

Avoid triggers to prevent flares of AD. These include soaps, shampoos, shower gels and bubble baths, prickly or rough clothing (including wool), overheating, overdressing, sweat, friction, direct contact with grass and sand, prolonged exposure to chlorine and salt water, or emotional stress.

### 2.6. Dysbiosis in Rosacea

Rosacea is a chronic inflammatory disease, which typically presents with facial flushing, persistent centrofacial erythema, telangiectasia, and inflammatory pustules. The relationship between dysbiosis and rosacea is thought to involve several organisms, namely, *Demodex* spp., *Bacillus oleronius*, *S. epidermidis*, and *Cutibacterium acnes* [50]. Demodex are a family of sebum-consuming mites (obligate human ecto-parasites), which are typically found in facial regions classically associated with rosacea. In cases of rosacea, skin samples have demonstrated higher frequencies of the demodex mite species when compared to control skin [7,51]. Research has also proposed that *Demodex* mites and their associated bacteria upregulate proteases that are linked to the further dysregulation of the cutaneous innate immune response [9]. The pathophysiology of this disease is a complex interplay, which interacts with barrier dysfunction and can lead to a decreased tolerability to skincare products in this patient cohort.

Whilst erythematotelangiectatic rosacea and seborrhoea are often treated with topical vasoconstrictors, pulse dye laser/intense pulsed light or topical retinoids respectively, the papulopustular flares of rosacea are associated with dysbiosis. Rosacea responds well to topical metronidazole 0.75% for 12 weeks but often recurs. Topical ivermectin 5% cream, a topical antiparasitic ointment, has been used in studies to decrease the burden of demodex mites, with clinical improvement in rosacea [49,52,53]. *Bacillus oleronius*, a Gram-negative non-commensal bacterium, has been isolated from *Demodex* mites and has been proposed as a possible inflammatory trigger in rosacea mediated through neutrophil activation [54]. Studies have demonstrated that this Gram-negative bacterium is susceptible to many antibiotics in conjunction with other therapies (Table 2) used to treat rosacea, and may well explain the link with dysbiosis [55]. While the previous mechanism of action for antimicrobial therapy in rosacea was presumed to be anti-inflammatory, there is a suggestion from these studies that antimicrobial effects are also exerted.

### 2.7. Dysbiosis in Hidradenitis Suppurativa 

Hidradenitis suppurativa (HS) is a chronic condition of the apocrine pilosebaceous unit with evidence of some role of dysbiosis [58]. As with rosacea, when compared with healthy controls, individuals with HS have lower counts of *Cutibacterium acnes*, possibly due to an associated disease-promoting disruption of the bacteriome, and a higher number of anaerobic Gram-negative bacteria [58,59]. Specific organisms may play a role, with lesions found to have a higher count of *Corynebacterium*, *Porphyromonas*, and *C. peptoniphilus* [24]. In addition, studies have reported an increased (relative) abundance of certain anaerobic bacteria (such as *Peptoniphilus* spp., *Prevotella*, and *Porphyromonas*) in lesional skin in hidradenitis suppurativa in contrast to control or non-lesional skin. The relative abundance of anaerobic bacteria and the increase in the diversity of bacteria were also shown to correlate positively with the severity of hidradenitis suppurativa [27].

**Figure 2:**
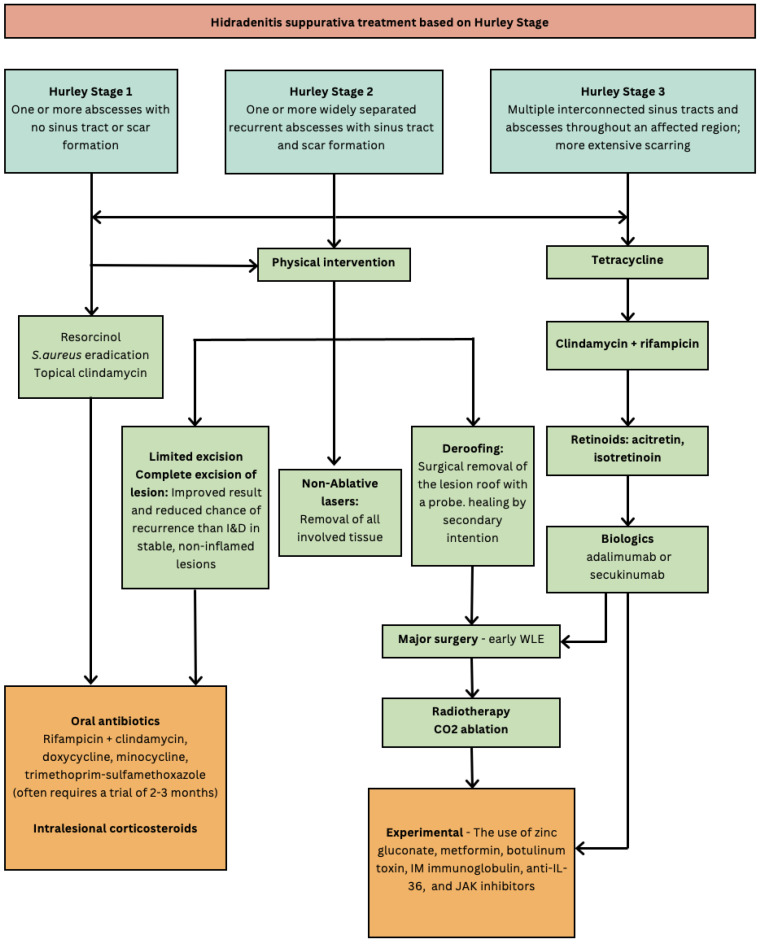
Hidradenitis suppurativa treatment based on Hurley Stage [32,45]. Flow diagram of treatment options for hidradenitis suppurativa based on Hurley Stage. WLE (wide local excision); I&D (incision and drainage); IM (intra-muscular).

The use of targeted antibiotics to induce HS remission in patients with syndromic disease forms has been documented and forms an important part of HS management (Figure 2). Syndromic forms associated with HS include PASH (pyoderma gangrenosum, acne conglobata, and suppurative hidradenitis) and PAPASH (pyogenic arthritis, pyoderma gangrenosum, acne, and suppurative hidradenitis) when HS presents as part of other inflammatory disorders. There are currently two biologic treatments that are FDA-approved for HS for treatment by a dermatologist. These are secukinumab (an interleukin-17A inhibitor) and adalimumab (an antibody targeting tumour necrosis factor-alpha) [60]. These biologic agents form management options for those with moderate-to-severe disease (Figure 2), and previous non-response/allergy/adverse reactions to two different courses of antibiotics, each for 3 months [61].

The formation of sinus tracks/tunnels and scarring in HS is likely part of the reason for an abnormal microbiome. The pathophysiology of hidradenitis suppurativa initially starts with follicular occlusion in areas of friction with skin rubbing on skin (e.g., axillae, groin) in addition to the hyperkeratinisation and dilatation of the follicles [62]. From here, bacteria and keratin are released into the dermis following the rupture of the follicle. Abscess formation can then be seen with profuse inflammatory responses. Immune dysregulation in hidradenitis involves a variety of chemokines and cytokines including IL-17, TNF, IL-1 α/β, G-CSF, and IL-6 [63,64]. From here, tissue is damaged via recruited neutrophils and associated neutrophil extracellular traps, reactive oxygen species, the involvement of pro-inflammatory cytokines, and the activation of the complement cascade (e.g., C5a, C3a) from recruited macrophages. Overall, tunnels and the generation of epithelial strands form from the rupture of the follicular epithelium and matrix metalloproteinases (degrading enzymes). Targeting the microbiome in patients with HS is a logical therapeutic option, and a greater understanding of skin microbiota may very well lead to a more precise therapeutic approach.

## 3. Conclusions

Future novel work is needed to explore the skin microbiome in clearly defined infectious diseases, in skin diseases that are exacerbated by infection, e.g., eczema, and in skin diseases that are treated with long-term antibiotics for the presumed but currently poorly defined role of bacterial pathogenesis, e.g., hidradenitis suppurativa. Understanding normal skin flora will help define how microbial imbalance may be associated with skin disease and skin healing. This will be invaluable in populations with a high burden of skin disease (e.g., children living in remote Indigenous communities in Australia who have the highest reported rates of impetigo in the world [65]). Future studies could also have wider implications for health in terms of the skin–gut microbiome axis and an impact on systemic infection and disease states [66].

In addition, skin microbiome protocols could be utilised to evaluate microbial dysbiosis in dermatological conditions (e.g., atopic dermatitis, papulopustular rosacea, hidradenitis suppurativa), the changes in the skin microbiome with biological therapies such as dupilumab in atopic dermatitis, and the impact on the skin microbiome in healing and burns.

Once skin microbiome protocols are optimised, future research could also further investigate the interplay between the skin and gut microbiota. Recent research into the “gut–skin axis” shows that connections between the skin and gut microbiota can influence both an individual’s likelihood of developing AD and the severity of disease after onset [67]. In rosacea, there is conflicting evidence in relation to the role of *Helicobacter pylori* and rosacea. A recent meta-analysis did not find any improvement in the symptoms of rosacea with *H. pylori* eradication or any statistically significant association between rosacea and *H. pylori* infection [68].

Future research will be able to utilise optimised skin microbiome protocols in order to further evaluate the composition of healthy/normal skin. Further research using culture-independent techniques is needed for conditions involving microbial dysbiosis (e.g., periorificial dermatitis, papulopustular rosacea, hidradenitis suppurativa). Temporal shifts in the composition of the skin microbiome have been described in skin conditions such as atopic dermatitis [26]. Future research aimed at understanding immune responses to certain bacteria as well as therapeutic agents to target pathogens and dysbiosis may offer novel treatment ideas for specific dermatological conditions. To understand these conditions better, we need improved techniques that extend our understanding of microbiology, from routine cultures to molecular techniques where the bacterial DNA signature can be determined.

Future studies could use an intensive bacterial ‘culturomic’ approach to isolate difficult-to-culture bacteria and to establish a repository of bacterial isolates, which we will use to try to understand why some bacteria are associated with disease and others with healthy skin. Developing high-quality protocols for the collection and testing of samples for skin microbiome analysis would yield unique resources globally, representing the leading edge of this emerging field.

It is evident that a lack of awareness about the diversity of the skin microbiome and poor sampling has hindered our progress in understanding the role of dysbiosis in inflammatory skin disease. Further research is required to discern the impact of the therapeutic actions of antibiotics and topical probiotics on the skin microbiome in inflammatory skin conditions. There is, however, great potential in that these individualised treatments in AD, rosacea, and HS may translate into targeted antimicrobial care, enhancing our future management of such dermatological conditions. 

## Figures and Tables

**Table 1:** Management of atopic dermatitis based upon severity [32,50].

Mild	Moderate	Severe
Emollients: Apply moisturiser at least daily in thick layer. If unresponsive to use of regular moisturiser, recommend mild–moderate-strength topical corticosteroids: treat until clear then reduce frequency. Research suggests that daily application of some topical corticosteroids is adequate; however, topical corticosteroids twice daily is usually recommended for AD. To prevent recurrent skin infections: dilute bleach baths (details below).	Emollients: Apply moisturiser at least daily in thick layer. Moderate-strength topical corticosteroids, i.e., methylprednisolone aceponate 0.1%, for use until skin is pruritus-free and smooth, then reduce gradually to minimum frequency that allows skin to be inflammation-free. Facial or eyelid dermatitis maintenance (age > 3 months): pimecrolimus 1% cream recommended for patients who have not had satisfactory control with use of intermittent topical corticosteroid or where topical steroid is contra-indicated. Consider referral to dermatologist if no improvement. To prevent recurrent skin infections: dilute bleach baths (details below). Oral antibiotics (e.g., cephalexin)—if clinically impetiginised, or anti-virals if Herpes Simplex is present.	Emollients: Apply moisturiser at least daily in thick layer. Potent topical corticosteroids (such as betamethasone dipropionate 0.05%) to affected areas on trunk and limbs. If non-responsive or symptoms persist while on potent topical steroids, refer to dermatologist. To decrease severity during flares, wet wraps with topical corticosteroids are recommended. Immune modulation with phototherapy, methotrexate, ciclosporin, mycophenolate, and azathioprine may be indicated in severe disease (refer to dermatologist). New management options such as dupilumab and JAK inhibitor, upadacitinib, can be accessed via dermatologists in patients meeting PBS criteria.

**Table 2:** Treatment options for rosacea subtypes (Australia/New Zealand Algorithm) [56] (reproduced with permission on behalf of the Australasian Medical Dermatology Group). Rademaker M. Medical Management of Rosacea—an Australian/New Zealand Medical Dermatology narrative. Presented at The Australasian College of Dermatologists 55th Annual Scientific Meeting; 28 May 2023; Sydney, Australia [57].

Treatment Options for Rosacea Subtypes								
Phenotype	Erythema			Papules and/or Pustules			Phyma	
	Transient	Persistent	Telangiectasis *	Mild	Moderate *	Severe *	Inflamed	Non-Inflamed
**Starting Rx**	Start one of topical brimonidine gel or oxymetazoline cream	Start one of topical brimonidine gel or oxymetazoline cream	Trial one of IPL Laser RF	Start one of topical azelaic acid ivermectin metronidazole	Start one of topical azelaic acid ivermectin metronidazole	Start one of Doxycycline or low-dose isotretinoin *	Start one of doxycycline or low-dose isotretinoin *	Ablative laser *
Inadequate response at 3/12 *	Add in oral β-blocker or clonidine	Add in a physical therapy *: IPL Laser RF Consider BoNTA *	Try a different physical therapy *: IPL Laser RF	Add in another topical: azelaic acid or ivermectin or metronidazole Consider topical BPO or retinoid	3/12 of doxycycline	Low-dose isotretinoin * or hydroxychloroquine * or RF *	RF *	Surgical curettage * or RF *
**Next step**	Consider treating low-grade inflammation with low-dose isotretinoin * or hydroxychloroquine for 12 months *			Consider systemic Rx	Low-dose isotretinoin * RF *	Oral ivermectin or short-course systemic steroids * or dapsone *		
**Maintenance** **(12 months)**	Continue topical Rx if it was effective, repeat physical therapy when appropriate			Switch to topical Rx if possible, or continue low-dose isotretinoin *			Continue low-dose isotretinoin *	

IPL (intense pulsed light); RF (fractional radiofrequency); Rx (therapy/medication); BoNTA (Botulinum toxin type A); BPO (benzoyl peroxide). * Referral to a dermatologist recommended.

## Data Availability

No specific data were required in the preparation of this manuscript.
